# Reimagining Innovation Amid the COVID-19 Pandemic: Insights From the WISH Innovation Programme

**DOI:** 10.3389/fpubh.2021.678768

**Published:** 2021-06-07

**Authors:** Maha El Akoum, Mahmoud El Achi

**Affiliations:** World Innovation Summit for Health, Qatar Foundation, Doha, Qatar

**Keywords:** innovation, healthcare innovation, cost efficiency, public health, competition

## Abstract

The World Innovation Summit for Health (WISH) hosts two innovation competitions as part of its biennial healthcare conference. During the COVID-19 pandemic, WISH received more than 350 applications for both competitions, of which 31 were shortlisted to showcase at the WISH 2020 virtual summit. Of the 31 showcasing innovations, 11 (35.5%) had suggested an alternative use to their innovation as a contribution to the global fight against COVID-19. As such, this article explores the apparent and urgent need for the repurposing of healthcare innovations to reduce the costs and time associated with the conventional approach, in order to best respond to the demands of the global pandemic.

## Introduction

The world is currently facing one of the greatest social and economic challenges since World War II (1). The key to overcoming the COVID-19 pandemic lies in finding innovative and effective treatments for the disease, further emphasizing the need for an outside-in approach to healthcare innovation (2). The lengthy, conventional path to the diffusion of innovation has been complemented, and in some cases replaced, with an ultrafast approach that focuses on the repurposing of knowledge, ideas, and available technologies in order to provide rapid solutions to the crises at hand (3). In this context, we explore how existing startups and ventures have responded to the lack of available resources and increased pressure on healthcare systems worldwide by reimagining their initial purpose to navigate COVID-19. Through the thorough examination of applications received and shortlisted for the World Innovation Summit for Health (WISH) Innovation Programme, we consider the innovative technologies that have evolved to react efficiently and effectively to this global health emergency.

## Wish Innovation Programme

### About WISH

WISH is a biennial global gathering of healthcare leaders, policy makers, academics, and innovators based in Doha, Qatar, and charged with building a healthier world through global collaboration. As well as presenting panel discussions and presentations, WISH commissions and undertakes research and also offers a platform for showcasing innovation in health. At the core of the aim of showing health innovation are two innovation competitions, open to entrepreneurs toward the start of their product development journey.

### WISH Innovation Competition 2020

In March 2020, days before the coronavirus outbreak was declared a pandemic by the World Health Organization (WHO), WISH launched two rebranded healthcare innovation competitions: the “Spark Competition” and “Booster Competition.” These competitions provided talented and striving entrepreneurs with a platform to promote ideas and products aimed at improving the delivery and quality of healthcare. Applications to the competitions remained open until July 31, 2020.

The WISH Innovation Spark Competition was aimed at entrepreneurs who were seeking to solve healthcare challenges by developing and validating a scalable project. These were typically early-stage innovations or startups. The WISH Innovation Booster Competition was aimed at already existing healthcare solutions with innovators that were looking to scale up and grow. The competitions were open to local and international applicants, aligning with WISH's mission to build a healthier world through global collaboration. Successful applicants for both competitions were required to meet certain criteria, such as an evidence-based market need for the proposed innovation, and demonstrated potential for sustainable future growth.

The panel of judges included healthcare innovation experts, investors, innovation hub representatives, and scientists.

Among its local partners are Qatar Foundation's Qatar Science and Technology Park, Hamad Bin Khalifa University Innovation Center, Qatar Business Incubation Center founded by Qatar Development Bank and Qatar Research, Development and Innovation Council. Global hubs include the United Kingdom–based BMJ New Ventures; the HAG Group, a Brazilian innovation center; Portuguese innovation center Beta-I; and The Helix Centre in the United Kingdom.

## Methods

We examined the applications received for the WISH Innovation Programme to identify which of the applicants had suggested an alternative use of their innovation for COVID-19 diagnosis or treatment of symptoms. This involved the sorting and filtering of applications through the report function of Apply by SurveyMonkey, exporting this to Microsoft Excel and filtering again manually by mention of “COVID.” Upon detailed review, two innovations were excluded as the applicant had not mentioned an alternative use or potential use of the product for COVID-19. Ten innovations that were created for the sole purpose of COVID-19 were also excluded so as to focus on the theme of innovative repurposing. For the purpose of this article, only the shortlisted innovations showcased at WISH and that had highlighted and provided evidence for a potential use for COVID-19 either through the online application portal or through personal communication after the summit were considered.

## Results

A total of 350 applications were received via the application portal and through email communication. Of those 350, 49 (14%) had suggested a potential for use for COVID-19. Of the 31 innovations that were shortlisted to showcase at WISH 2020, 11 (35.5%) had reported an alternative potential use for COVID-19. The innovations were split into six different categories based on type, function, and mode of care or service delivery. These categories included artificial intelligence (AI) solutions, digital/app-based solutions, medical/scientific equipment, community-based/social solutions, education and training, and telemedicine. Table 1 lists and describes these innovations in some detail and explores their original function or purpose, and how they were repurposed or could potentially be repurposed, to provide solutions to different aspects of the COVID-19 pandemic. Webpage links have been provided for each of the innovations for more information.

**Table 1:** Shortlisted innovations and their potential to help with COVID-19 pandemic.

**Name of innovation**	**Description of its main purpose**	**COVID-19 implication**
**Artificial intelligence**		
Kalya™	Kalya is an artificial intelligence solution focused on non-pharmacological interventions such as physical interventions, psychological interventions, nutritional Interventions, and digital interventions. This helps address the major health challenges of today and tomorrow (cancer, diabetes, aging, strengthening the immune system, lifestyle). Kalya's expertise enables the design of dedicated digital solutions from the indexing of scientific literature to the exploration and analysis of data to support the decisions of health professionals.	Complementary, non-pharmacological interventions provided digitally are extremely relevant in the treatment context of COVID-19. Especially as the availability of new research findings on interventions for COVID-19 is being updated almost daily, Kalya can help use these new emerging data to help support the decisions of frontline workers—saving them time and providing the patients with the best possible care.
iLoF™: cloud-based library of disease biomarkers and biological profiles	iLoF is a cloud-based library of disease biomarkers and biological profiles based in Oxford, United Kingdom. It uses AI and photonics to build a cloud-based library of diseases and their biomarkers and biological profiled to drastically reduce the cost and time of drug discovery and drug repurposing. Supported by the University of Oxford and Microsoft Ventures, iLoF is being used to find solutions for one of the biggest epidemics of our time, Alzheimer disease. It is also being used to find potential treatments for digestive cancers, stroke, and infectious diseases.	An indicated alternative potential use for iLoF is that it can be used to generate clinical output, operating as a forecast tool that predicts the severity of symptoms for COVID-19 patients.
Botkin™	Botkin.AI is a software platform based on artificial intelligence technologies for the analysis of different types of radiological studies. Main purposes include early detection of lung malignant neoplasms, early detection of breast cancer, detection of lung non-oncological pathologies (tuberculosis, pneumonia, etc.), and COVID-19 detection. Botkin.AI has commercial and pilot integrations in four countries and possesses the best result in terms of lung oncology detection in the world.	Botkin can be used for detection of COVID-19 through radiological analysis. Botkin.ai's X-Ray Analysis function is intended to provide assistance to a radiologist in order to help find pathological changes in chest x-rays. Early detection of medically actionable pathological changes can potentially lead to early hospitalization and treatment and, in turn, a decrease in mortality rate.
Pandexit™	Pandexit is a software that uses an algorithm to predict the evolution of any pandemic weeks in advance. The revolutionary algorithm is used to build a high-granularity model of any pandemic for an entire country. The software then generates the policies needed to fight and end the pandemic, both saving lives and protecting the economy.	Pandexit has successfully been deployed in three countries; it has been used by the special teams in charge of fighting the COVID 19 pandemic to simulate scenarios, model a number of policies, discover their effects on the spread of the pandemic inside the population, and select the most efficient way to save lives and limit the damages to the economy. At the vaccine rollout stage of the pandemic, Pandexit allows the decision makers to model an increasing number of vaccinated people in specific categories of the population and take new variables of the virus into account. Pandexit models are increasingly accurate due to the emerging data on COVID-19 and its variants. It can evaluate the efficiency of an implemented curfew and a lockdown and can predict and recommend the exact number of days needed to reach a specific target.
**Digital/app-based**		
Dorothy.app™	Dorothy.app is a monitoring system created for dementia patients. Dorothy.app transforms a standard walker into an augmented reality-based navigation assistant for those with dementia using a tablet computer. It allows family to both communicate with and remotely check the well-being of their loved one. Dorothy transmits real-time location data to family members allowing easy communication, maximizing social networks, and care collaboration. It can detect deterioration in general health.	Dorothy increases the independence of those with dementia while also being able to revolutionize how support and monitoring are delivered. It also supports the “recovery” phase of the COVID-19 response, particularly in often quarantined facilities. As language is also often impaired, the telemetry data can initially inform family and clinicians about activity and provide alerts when changes in such data suggest an deterioration in general health allowing for more holistic remote care.
**Medical**/**scientific equipment**		
AMSU™: The Airway Medical Suction Unit	AMSU looks like a sports bottle. It incorporates a Venturi that turns positive pressure from a small can of chlorofluorocarbon-friendly gas into negative pressure (suction). Through careful selection of gas (positive) pressure and Venturi size, it is possible to incorporate an effective laryngeal suction device into the cap of the “sports bottle.” It is used to clear blocked airways in an emergency. It also helps clear sputum or vomitus on a regular basis in people with chronic brain injury conditions.	CAMSU™, the COVID-19 Airway Medical Suction Unit, is a wall-mounted suction unit that was developed as part of the pandemic response. It can be used to clear some COVID-19 symptoms by preventing sputum from incubating in the laryngeal area and traveling to the lung cavity. This, in turn, would improve outcomes and prevent some ICU admissions for sedation/intubation/ventilation, saving money and freeing up hospital beds.
We care solar™	Solar suitcase is a compact solar electric system that provides power generation and medical appliances. Some of its features include high-efficiency, water-resistant, long-lasting (70,000 h) LED lights designed for surgical procedures; 12-V DC and 5-V DC power ports for mobile communication; an electronic fetal heart rate monitor; an electronic infrared thermometer, tablets, and e-readers equipped with educational materials.	The no-touch infrared thermometer has been used in COVID-19 assessments. Improved phone charging ensures that emergency referrals happen when needed, especially given that the target population is rural, improvised communities in Africa and Southeast Asia.
QABY biotech™	QABY is a Qatar-based biotech startup that encompasses novel antibodies, innovative assays, unique biomarkers, and the evidence-based knowledge on how to tackle neurodegenerative diseases. QABY can facilitate the early detection of neurodegenerative diseases, assess treatment responses, and monitor disease progression. QABY's key activities include research and development of tools and assays and discovery of new disease targets, providing services through participation in clinical trials, and selling antibodies and kits directly to end users.	QABY received an innovation grant from Hamad bin Khalifa University Innovation Center to develop a serology test for COVID-19. The blood test was successfully completed within 6 months, and the data are strongly correlated with data generated using other well-established technologies, further validating the robustness and usefulness of the in-house kit. Currently, the innovator is also finalizing the development of anther test for COVID-19, named neutralization assay to be added to the QABY portfolio.
**Education and training**		
Medics.Academy™	Medics.Academy is a technology-enhanced learning and education platform. It uses advanced technologies to scale programs across countries and to reach large-scale audiences of healthcare workers.	Medics.Academy was commissioned by the WHO to help deliver an education and training program to support the WASH agenda as part of the COVID-19 response.
**Community based solution**		
Charly™	Charly is a speech assistant/speech recognition device that was designed to promote independent communication between people with hearing impairments. Charly recognizes speech and converts it into text.	Charly has been helping people with disabilities at the Employment Center in Moscow to find jobs after having lost them (in some cases due to COVID-19). This has proven to be cheaper, faster, and more efficient than having an interpreter. The founders are also looking at ways to make Charly more useful for distant working. Examples could include help with university lectures for students studying remotely due to the restrictions brought about by the COVID-19 pandemic.
**Telemedicine**		
Bleepa™	Bleepa is a secure clinical messaging app that facilitates remote image-based communication between clinicians. It allows medical staff to view and discuss high- quality medical-grade imaging on mobile devices.	Since the onset of COVID-19, Bleepa has been helping senior consultants to guide frontline staff and manage cases remotely and safely without face-to-face interaction.

The most common category or field of innovations and technological solutions to suggest an alternative use for COVID-19 was AI, where 4 of the total 11 listed innovations were AI-based. The second most common category was medical/scientific equipment (3 of 11), with the remaining four categories including one innovation each.

## Discussion

The COVID-19 pandemic is the first coronavirus pandemic to have a global effect (4). The novelty, speed, and severity of the virus, along with the lack of time and resources available for an effective and timely response, have all been contributing factors behind the race to find innovative therapies and technologies that not only improve health outcomes, but also soften the social and economic impacts of the pandemic. COVID-19 has also revolutionized the way in which solutions are found, developed, and deployed, redefining what it means to innovate. The conventional approach of extensive testing and trialing of new technologies, drugs, and processes has been replaced with a more “frugal” and efficient response that is focused on repurposing technologies and ideas that already exist (2, 3).

This has been evident in the outcome of the WISH innovation competitions whereby more than a third of the shortlisted applicants have demonstrated potential for their innovation to be used in the fight against the COVID-19 pandemic. This reemphasizes the notion that solutions to some complex problems may arise from rather unconventional sources—especially in the middle of a global crisis. Given the size of the crisis at hand, it is imperative that we remove regulatory barriers and facilitate this cost-effective and timesaving approach to healthcare delivery. In order to encourage this, an open innovation culture must be adopted whereby the innovation process involves purposive knowledge flow between organizational boundaries (5).

### Repurposing in the Pharmaceutical Industry

In the pharmaceutical industry, drug repurposing has been practiced for years. While *de novo* drug development requires substantial financial investments and years of experimentation, drug repurposing allows developers to cut time, cost, and risk by using AI and computational biology to find additional therapeutic uses of drugs that have previously been through these rigorous safety trials and are already on the market (6–8). Traditional methods of drug development require sometimes up to 15 years' worth of preclinical research, clinical studies, safety reviews, US Food and Drug Administration (FDA) review, and post-market monitoring. Many repurposed drugs have already been approved by the FDA saving both time and millions of dollars (9, 10). There have been several successful examples of drugs that have been repurposed across different fields such as diabetes and oncology (11). In the case of the COVID-19 pandemic, as vaccine development and rollout take months, different drugs are being repurposed in order to help alleviate symptoms and prevent deaths. The efficacy of these drugs in achieving favorable outcomes in patients with COVID-19 is still being studied (12–15).

### Repurposing in Healthcare and Service Provision

Similar to what has been witnessed from the WISH innovation competitions, other examples of healthcare innovations being repurposed and deployed in the global battle against COVID-19 have been recently recorded in the literature. One example from the United Nations Development Programme Accelerator Lab in collaboration with the Rwandan Ministry of ICT and Innovation included the deployment of five antiepidemic robots to aid frontline workers to fight spread of the COVID-19 virus (16). These robots provided support in detection of the COVID-19 cases, including citizens returning from travel, as well as providing other services in the hospital setting.

Another large-scale example comes from India where railway coaches were converted to isolation wards for COVID-19 patients to meet the increased and unmet demand for hospital beds (17). Such innovative and resourceful tactics have been practiced by low- and middle-income countries for years and are generally praised for expanding access to care, while fostering a uniquely competitive environment for creating solutions that are increasingly sustainable (18).

Hospitals in their entirety have also been reshaped and repurposed in the fight against the COVID-19 pandemic, where around the world we have witnessed many mitigation strategies deployed at a healthcare system level and within the hospital setting. Examples from South Korea include stratification of patient care, the establishment of COVID-19 hospitals and dedicated COVID-19 emergency centers, the establishment of dedicated community facilities and emergency centers, and the assignment of respiratory care split hospitals (19). These were all applied effectively and have been key in tackling the crisis at hand given the strain on healthcare resources.

### The Use of AI During COVID-19

Health systems are coping with the explosive demand through the large-scale adoption of AI and digital operating models (20, 21). AI can be used in a number of significant applications in the fight against the COVID-19 pandemic. These applications range from early detection and diagnosis, treatment monitoring, contact tracing, projection of cases and mortality, development of vaccines and drugs, decreasing healthcare workers' workload, and disease prevention (22).

One example of AI being used for detection and diagnosis purposes includes Providence St. Joseph Health system in Seattle, whereby in partnership with Microsoft, Providence developed an online screening and triage solution. This tool could help detect and differentiate, at a rapid rate, the difference between a patient with COVID-19 and a patient with another, less threatening ailment (20). This same partnership, together with University of British Columbia, has also provided other AI-driven solutions to monitor the compliance and effectiveness of social distancing measures, as well as personal protective equipment (PPE) usage (23).

Similar to “Pandexit” mentioned in Table 1, another innovation that has also been deployed in the use of virus tracking is Metabiota. Metabiota, a company that provides services for the US Department of Defense and intelligence agencies, has been used to detect the risk of spread of disease, calculating its predictions from factors such as symptoms, availability of treatment, and mortality (24).

### The Rise of Telehealth During the COVID-19 Pandemic

The use of telehealth or telemedicine technologies to combat the current COVID-19 crisis, as well as the overall management of communicable diseases as a whole, is ideal. Telehealth solutions decrease the need for person-to-person contact, so for COVID-19 patients, or patients who are concerned about getting infected, telehealth can provide an effective alternative solution for remote assessment and delivery of care (25, 26).

Other advantages of telehealth include increased access and availability to specialists and subspecialists. These can sometimes be doctors in different hospitals, providing care in different regions. The Mount Sinai system, for example, leverages specialists across eight hospitals to provide emergency consultations and make referrals where needed (27). The limitation of these types of solutions is usually related to issues to do with payment, staffing, and credentialing. It is also argued that nurses, medical assistants, and physician assistants all contribute to the overall experience of in-person care provision, and telemedicine could not possibly replicate this (27).

Telemedicine can also take the format of a messaging app between doctors to facilitate discussions and shared experiences and recommendations when it comes to more challenging medical cases. Such is the case of “Bleepa,” one of the WISH 2020 shortlisted innovators mentioned in Table 1. Here the challenge is avoiding the leak of confidential and sensitive information, such as the patient's condition and medical history.

### Lessons From Other Industries

Other industries, sometimes with no clear links to the healthcare industry, are also following suit. Several firms, for example, have exapted their capacities and capabilities to produce PPE (28). In addition, a number of cosmetic brands have started manufacturing hand sanitizers to meet the increase in demand. These brands include the likes of L'Oreal and Nivea (29). Another example is Dyson, a producer of household items such as air purifiers, vacuum cleaners, and hair dryers. Dyson announced in March 2020 that it had initiated the design and production process of 10,000 ventilators, following the United Kingdom's National Health Services' predictions of an increased demand for ventilators amid the COVID-19 pandemic (30). This type of innovative cross-industry collaboration to respond to the global demand for healthcare is an approach to be applauded and encouraged.

## Conclusion

It is without a doubt that the COVID-19 pandemic has emphasized the need for an accelerated, cross-sectorial, and frugal approach to healthcare innovation. The innovation competitions at WISH further emphasized this need through direct response, where more than a third of the applicants suggested an alternate use for their innovation. While repurposing products is a practice that has been going on for years before the onset of the current pandemic, it has been mostly been practiced by other industries such as drug development. Solutions that make use of AI and digital-based technologies for delivery of services and care are especially helpful during a global crisis such as this one where social distancing plays a great role in stopping the spread of the virus. It is also evident that not all solutions must be high-tech in order for them to provide sufficient answers and deliver impact. Innovations such as AMSU, mentioned in Table 1, provide relatively low-tech, yet highly effective solutions. Finally, it is imperative that lessons are learned from this pandemic at a global level and that this fast and frugal method of responding to an increase in demand, especially in the time of crisis, becomes mainstream and runs in parallel to the more traditional approach to innovation and product development. In order for repurposing to become mainstream practice in healthcare innovation, there is a need for systems worldwide to facilitate this through the adoption of open innovation mechanisms.

## Data Availability Statement

The raw data supporting the conclusions of this article will be made available by the authors, without undue reservation.

## Author Contributions

MahaE wrote the draft with input from MahmE. Both authors contributed to the article and approved the submitted version.

## Conflict of Interest

The authors declare that the research was conducted in the absence of any commercial or financial relationships that could be construed as a potential conflict of interest.
